# Multiagent Large Language Model Framework for Psychotherapy Fidelity Assessment in Motivational Interviewing and Cognitive Behavioral Therapy Training: Cross-Sectional, Simulation-Based Evaluation Study

**DOI:** 10.2196/92964

**Published:** 2026-08-18

**Authors:** Mohammad Amin Kamaleddin, Mina Mirjalili, Reza Barzegar, Nghia Trung Le, Zachary Cote, Olga Winkler, Lisa Burback, Yanbo Zhang, Osmar R Zaiane, Candice Monson, Divya Sharma, Sri Krishnan, Andrew J Greenshaw, Richard Zeifman, Peter Selby, Venkat Bhat

**Affiliations:** 1 AI for Mental Health Program, St. Michael’s Hospital, Unity Health Toronto, Toronto, ON, Canada; 2 Temerty Centre for Artificial Intelligence Research and Education in Medicine, University of Toronto, Toronto, ON, Canada; 3 Campbell Family Mental Health Research Institute, Centre for Addiction and Mental Health, Toronto, ON, Canada; 4 Adult Neurodevelopment and Geriatric Psychiatry Division, Centre for Addiction and Mental Health, Toronto, ON, Canada; 5 Department of Psychiatry, University of Alberta, Edmonton, AB, Canada; 6 Neuroscience and Mental Health Institute, University of Alberta, Edmonton, AB, Canada; 7 Department of Computing Science, University of Alberta, Edmonton, AB, Canada; 8 Alberta Machine Intelligence Institute, Edmonton, AB, Canada; 9 Department of Psychology, Toronto Metropolitan University, Toronto, ON, Canada; 10 Department of Mathematics and Statistics, York University, Toronto, ON, Canada; 11 Department of Electrical, Computer, and Biomedical Engineering, Toronto Metropolitan University, Toronto, ON, Canada; 12 NYU Langone Center for Psychedelic Medicine, Department of Psychiatry, NYU Grossman School of Medicine, New York, NY, United States; 13 Center for Psychedelic Research, Department of Brain Sciences, Imperial College London, London, United Kingdom; 14 Department of Psychology, The New School for Social Research, New York, NY, United States; 15 Department of Family and Community Medicine and Dalla Lana School of Public Health, University of Toronto, Toronto, ON, Canada; 16 Department of Psychiatry, University of Toronto, 250 College Street, 8th FloorToronto, ON, M5T 1R8, Canada, 1 416-360-4000 ext 76404

**Keywords:** psychotherapy, artificial intelligence, AI, large language models, motivational interviewing, cognitive behavioral therapy, treatment fidelity, clinical competence

## Abstract

**Background:**

Psychotherapy training is difficult to scale because manual rating of motivational interviewing (MI) and cognitive behavioral therapy (CBT) sessions is time-intensive, requires trained raters, and is subject to rater variability. Large language models (LLMs) may support simulation-based training and rubric-guided scoring, but early-stage evidence is needed before such systems can be applied to real learners.

**Objective:**

This study aimed to conduct a simulation-based evaluation of a multiagent LLM framework for generating and scoring stylized MI and CBT training encounters.

**Methods:**

We conducted a cross-sectional evaluation of a multiagent framework comprising Student, Patient, Evaluator, and Feedback agents. The Student agent conducted synthetic MI or CBT encounters with Patient agents derived from structured profiles. The Evaluator agent scored transcripts using study-specific MI and CBT scoring forms. Internal discrimination was tested across prompt-engineered novice, intermediate, and expert Student agent profiles using 133 MI and 102 CBT Patient profiles. Preliminary external grounding was assessed using 133 annotated motivational interviewing (AnnoMI) transcripts with coarse, metadata-derived, high/low session-level quality labels. Agreement with a pragmatic human-rater benchmark was evaluated using 16 independent human raters per modality. Criterion-level comparisons used paired Wilcoxon signed-rank tests with Benjamini-Hochberg false discovery rate correction at *q*<0.05. Agreement analyses used intraclass correlation coefficients (ICCs) with bootstrap 95% CIs. A secondary prompt-augmentation sensitivity analysis tested whether appending criterion-referenced feedback text to the novice Student-agent prompt shifted subsequent Evaluator-assigned scores.

**Results:**

Evaluator scores increased across prompt-defined Student-agent competence levels. For MI, overall mean scores increased from 1.18 (95% CI 1.16‐1.20) for novice profiles to 1.75 (95% CI 1.69‐1.81) for intermediate profiles and to 3.57 (95% CI 3.48‐3.66) for expert profiles. For CBT, overall mean scores increased from 0.83 (95% CI 0.78‐0.88) to 2.18 (95% CI 2.10‐2.26) and 4.38 (95% CI 4.28‐4.48), respectively. Criterion-level planned contrasts were significant after false discovery rate correction. On AnnoMI transcripts, Evaluator scores aligned with metadata-derived high/low session labels, with 91.7% classification accuracy at the prespecified threshold. Human interrater reliability was an ICC(2,1) of 0.866 for MI and 0.769 for CBT. Evaluator-vs-human-consensus agreement was an ICC(2,1) of 0.959 for MI and 0.934 for CBT. In the secondary prompt-augmentation analysis, overall MI scores shifted from 1.18 to 1.44, and CBT scores shifted from 0.83 to 1.01.

**Conclusions:**

This proof-of-concept study suggests that a rubric-guided multiagent LLM framework can score stylized synthetic MI and CBT transcripts along expected prompt-defined competence gradients and align with preliminary external and human-rater benchmarks. The study is innovative in separating synthetic learner, patient, scoring, and feedback-generation roles within a single simulation workflow, extending prior LLM work from plausible dialogue generation toward rubric-linked scoring. The findings support further development of scalable simulation tools for psychotherapy training research. Prospective studies with human trainees, standardized or real patients, and formal psychometric testing are required.

## Introduction

Providing opportunities to practice clinical counseling or psychotherapy skills before seeing patients is foundational to psychiatric and psychological education [1]. Traditional simulations using peers or trained standardized patients offer realistic yet controlled environments for skill acquisition and are particularly effective for practicing procedural and interpersonal skills [2-4]. However, simulation with standardized patients is resource-intensive to create, staff, and scale across cohorts, sites, and time zones, demanding substantial educator time and expertise. These challenges include variability in rater expertise and feedback quality, as well as the high cost of sustained supervision [5]. These constraints are especially salient for psychotherapy-focused encounters, where trainees must combine theoretical understanding of a therapeutic approach with repeated, coached practice to develop skillful listening, empathy, and collaborative problem-solving [6].

Recent reviews of virtual simulation in health professions education indicate that digital patient tools can support repeated, standardized communication-skills practice, but that evidence remains heterogeneous and many systems are still evaluated mainly on usability, satisfaction, or perceived realism rather than objective competency outcomes [7]. The rapid emergence of large language model (LLM)-based virtual patients has intensified both this opportunity and this concern: recent scoping-review evidence suggests that LLM-based virtual-patient research is expanding quickly but remains early-stage, with inconsistent assessment designs and limited use of validated tools or objective learning outcomes [8]. For psychotherapy education, this gap is especially important because competence is not captured by plausible dialogue alone; trainees must demonstrate model-specific behaviors such as reflective listening, evocation, autonomy support, collaborative agenda setting, and guided discovery [9].

Motivational interviewing (MI) [10] and cognitive behavioral therapy (CBT) [11] are evidence-based interventions taught across mental health training programs. Both have structured, model-anchored therapist behaviors, making them relevant candidates for AI-based tools that support rubric-guided fidelity assessment. Observer-rated fidelity and competence instruments, such as the Motivational Interviewing Treatment Integrity (MITI) [12] and Motivational Interviewing Skill Code (MISC) [13] for MI and the Cognitive Therapy Rating Scale (CTRS) [14] for CBT, provide structured scoring anchors for rating therapist behaviors and session structure from transcripts or recordings. However, applying these scoring tools consistently is labor-intensive and susceptible to rater drift.

Automating psychotherapy fidelity assessment has therefore become an important target for computational behavioral science [15]. Recent MI-focused work has shown that AI models can classify counselor and client MI behaviors in chat-based counseling data and may support scalable coding, adherence monitoring, and personalized feedback [16]. Other recent work has used LLM-based and sequential modeling approaches to estimate MI session quality, reinforcing the potential of automated transcript-level assessment while also underscoring the need for further validation against expert and field-collected data [17].

LLMs add a complementary capability to prior virtual-patient and automated-coding approaches: they can generate interactive patient dialogue, instantiate different learner or patient roles, and produce structured explanations or feedback within a single workflow [18-21]. Agentic designs use multiple specialized agents to simulate roles, critique outputs, and enforce constraints, enabling scalable practice through role separation, cross-agent checking, and standardized evaluation workflows with feedback aligned to therapeutic model criteria [22-24]. Recent LLM-based simulated-patient systems and agentic frameworks have shown feasibility for medical education, including scalable patient simulation, communication-skills practice, and automated feedback [25]. However, many such systems remain oriented toward history taking, clinical reasoning, or generic communication practice rather than psychotherapy-specific fidelity assessment [26,27]. In behavioral health, recent scholarship has emphasized that LLMs could support psychotherapy training and research, but that this is a high-stakes domain requiring transparent rubrics, careful evaluation, human oversight, and attention to safety, bias, and the distinction between superficially empathic language and model-concordant clinical practice [28]. Emerging psychotherapy-specific studies have begun to evaluate AI-generated MI patient simulations and LLM-supported counseling feedback [29], but evidence remains limited regarding rubric-linked scoring sensitivity, agreement with human-rater benchmarks, feedback alignment, and evaluation across more than one psychotherapy modality.

To address these gaps, we conducted a preclinical, simulation-based evaluation of a rubric-guided, multiagent LLM framework comprising (1) a Patient agent that enacts structured Patient profiles through case-consistent responses; (2) a Student agent that conducts the synthetic session; (3) an Evaluator agent that scores Student-agent behavior against explicit criteria; and (4) a Feedback agent that generates criterion-referenced narrative recommendations. MI scoring used a study-specific MITI or MISC-derived global-rating form, and CBT scoring used a CTRS-derived form. MI Patient profiles were generated from a manually constructed template across common behavior-change contexts, whereas CBT Patient profiles were reformatted from the *Diagnostic and Statistical Manual of Mental Disorders, Fifth Edition* (*DSM-5*) Clinical Cases into a standardized Patient-agent prompt structure [30]. Annotated motivational interviewing (AnnoMI) [31] was used separately for a preliminary external comparison of Evaluator scores with coarse metadata-derived MI session-level quality labels. These simulations were intended as stylized benchmark examples for early-stage system evaluation, not as evidence that LLM-generated sessions reproduce the ecological complexity of real psychotherapy encounters.

The aim of this study was to conduct a preclinical, cross-sectional, simulation-based evaluation of a rubric-guided, multiagent LLM framework for psychotherapy training and fidelity assessment in MI and CBT. The primary objective was to determine whether the Evaluator agent could discriminate among prompt-defined novice, intermediate, and expert Student-agent performance across standardized synthetic Patient-agent encounters. We hypothesized that Evaluator-assigned scores would increase monotonically across these competence levels. Secondary objectives were to assess whether Evaluator-agent scores aligned with external AnnoMI session-level MI quality labels, to estimate agreement between Evaluator-agent scores and a pragmatic human-rater benchmark, and to examine whether appending criterion-referenced feedback to novice Student-agent prompts would shift subsequent Evaluator-assigned scores. Exploratory analyses examined Student-agent response-consistency metrics across prompt-defined competence levels.

## Methods

### Study Design

This was a preclinical, cross-sectional, simulation-based evaluation of a multiagent LLM framework for psychotherapy training and fidelity scoring. The study used synthetic Patient-agent encounters and prompt-defined Student-agent profiles to evaluate (1) internal scoring sensitivity across prompt-defined competence levels, (2) preliminary external alignment with AnnoMI session-level labels, (3) agreement with a human-rater benchmark, (4) secondary prompt-augmentation sensitivity after feedback text was appended to the novice Student-agent prompt, and (5) exploratory Student-agent response-consistency metrics. Where applicable, reporting of the AI system, evaluation setting, and human comparators followed the DECIDE-AI (Developmental and Exploratory Clinical Investigations of Decision Support Systems Driven by AI) guideline for early-stage evaluation of AI decision-support systems (Checklist 1) [32].

### Setting

Simulations and analyses were conducted computationally using Python or a LangGraph (LangChain) workflow. Human raters completed transcript-rating tasks remotely using synthetic or deidentified transcripts.

### Inclusion and Exclusion

AnnoMI transcripts were included if they had an available high or low session-level quality label. Human-rater data were included if the rater completed all assigned transcript-rating fields.

### Sampling Procedures

The MI and CBT Patient-agent profile sets were assembled purposively to provide broad coverage of behavior-change topics and diagnostic presentations for synthetic simulation, rather than to represent an epidemiologic or clinical population sample. MI Patient-agent profiles were generated using a manually written template across common MI-relevant behavior-change contexts. CBT Patient-agent profiles were reformatted from *DSM-5* Clinical Cases into standardized Patient-agent prompts. AnnoMI transcripts were included as a publicly available external comparison dataset. Physician raters were recruited purposively based on their clinical experience with MI and CBT. Additional independent human raters were recruited through Prolific using convenience sampling from the participant pool. Eligibility criteria required English as a first language.

### Sample Size, Power, and Precision

The study size was determined pragmatically based on the available Patient-agent profile set, the eligible AnnoMI transcripts with session-level labels, and the feasibility of obtaining human ratings. We analyzed 133 MI Patient-agent profiles, 102 CBT Patient-agent profiles, 133 AnnoMI transcripts, and 10 selected sessions per modality for human rating by 16 raters. Precision was summarized with 95% CIs for key agreement metrics, classification metrics, and mean estimates.

### Participant Characteristics

No real patients or real learners participated in the simulation experiments. Human involvement was limited to independent transcript rating. For each modality, 16 human raters scored the selected transcripts. The rater panel included 3 practicing physicians with clinical experience using MI and CBT, with a median of 5 (IQR 3-5) years of relevant clinical practice. Thirteen additional independent human raters were recruited through Prolific. All raters provided informed consent and were compensated at a rate equivalent to US $20 per hour.

### Patient-Agent Profiles

For MI, we constructed 133 Patient-agent profiles from a manually written template informed by MITI [12] and MISC [13] concepts and common MI-relevant behavior-change scenarios, including alcohol use, smoking cessation, weight management, and medication adherence. One profile was manually written as a template specifying required sections: role-prompt field, demographic and presenting details, behavioral presentation, MI-relevant elements, mental-status cues, and simulation-prompt field. We then used single-example template prompting, meaning that the LLM was given one completed exemplar profile, the required output fields, and instructions to generate additional Patient-agent profiles in the same structure. MI profile generation used GPT-4.1-mini. Demographic and clinical variation was introduced through prompt-specified variation in age, sex or gender, cultural background, presenting problem, readiness to change, sustain talk, change talk, and ambivalence (Table S1 in Multimedia Appendix 1).

For CBT, we constructed 102 Patient-agent profiles by reformatting cases from *DSM-5* Clinical Cases [30] into a standardized Patient-agent format using GPT-4.1-mini. Each profile included a case synopsis, behavioral presentation, symptom list, structured patient history, and diagnosis. The profile set was intended to provide broad diagnostic and cognitive or behavioral diversity for synthetic CBT simulations rather than a representative sample of CBT patients (Table S2 in Multimedia Appendix 1).

### AnnoMI External Comparison Dataset

We used 133 publicly available AnnoMI counseling transcripts for external comparison. AnnoMI includes expert utterance-level annotations; however, the present analysis did not use utterance-level behavior codes. Instead, we compared Evaluator-assigned global MI scores with the corpus-level high or low session quality labels, which are coarse, metadata-derived labels based on the original video context. This analysis was therefore treated as preliminary external grounding rather than as validation against expert fidelity coders.

### Measures and Covariates

Primary outcomes were criterion-level Evaluator scores within MI and CBT. MI scores were assigned across 9 criteria on a 1 to 5 scale using a study-specific MITI or MISC-derived global-rating form. CBT scores were assigned across 11 criteria on a 0 to 6 scale using a CTRS-derived scoring form [14]. No demographic or clinical covariates were modeled because Patient-agent profiles were synthetic and were not designed as a representative clinical sample.

### Evaluator Agent Rubric Construction

For MI, we created a study-specific MITI or MISC-derived rubric rather than applying the full MITI or MISC instruments. The purpose was to provide a compact global-rating form suitable for short simulated transcripts. Items were selected if they represented core MI fidelity constructs and could be judged at the transcript level as global performance domains. The 9 selected domains were Cultivating Change Talk, Softening Sustain Talk, Partnership, Empathy, Acceptance, Direction, Autonomy Support, Collaboration, and Evocation. MITI-derived domains were retained as global 1 to 5 ratings. MISC-derived domains were harmonized into the same 1 to 5 ordinal global-rating format using the source construct definitions as behavioral anchors. MISC behavior-count codes were not used as count variables.

The MI scoring form used a 1 to 5 global-rating scale for each criterion (1=“absent, clearly inconsistent with the construct, or strongly nonadherent”; 2=“minimal or mostly ineffective evidence”; 3=“partial, inconsistent, or mixed evidence”; 4=“generally competent and mostly consistent evidence”; and 5=“strong, sustained, and consistently adherent evidence across the transcript”). Item-specific anchor descriptions are provided in Table S3 in Multimedia Appendix 1.

For CBT, we used the 11 CTRS-derived domains using the original 0 to 6 structure (0=“poor or absent evidence of the competency”; 2=“limited or partially adequate performance”; 4=“good or competent performance”; and 6=“excellent, sustained, and well-integrated performance”). Scores of 1, 3, and 5 were used for intermediate judgments between adjacent anchors (Table S4 in Multimedia Appendix 1).

For both approaches, the Evaluator generated criterion-level scores linked to behavioral anchors, and the Feedback agent generated criterion-referenced narrative recommendations based on the transcript, scoring criteria, and scored evaluation output (Tables S5 and S6 in Multimedia Appendix 1).

### Narrative Feedback Generation and Alignment

The Evaluator agent assigned criterion-level scores. The Feedback agent then generated narrative recommendations using the transcript, scoring criteria, and the scored evaluation output. Feedback outputs were structured by criterion and included the assigned score, a transcript-grounded explanation, a short summary, and actionable recommendations. We treated narrative-feedback alignment as structural consistency between the numeric score, the criterion-specific explanation, and the recommendations provided. Representative aligned MI and CBT feedback excerpts are provided in Table S13 in Multimedia Appendix 1.

### Multiagent System and Model Configuration

We implemented a role-separated multiagent framework comprising Student, Patient, Evaluator, and Feedback agents to simulate MI and CBT encounters, score transcripts, and generate criterion-referenced narrative feedback. The system was implemented in Python version 3.11 and orchestrated using LangGraph to support stateful workflows. The Student and Patient agents used GPT-4.1-mini. The Student agent used a temperature of 0.5 to balance adherence to the prompt-defined competence profile with conversational flexibility, whereas the Patient agent used a temperature of 1.0 to allow greater variability in synthetic patient responses. The Evaluator agent used Gemini 2.5 Flash Lite with a temperature of 0.2 to promote more stable rubric scoring and to assign the scoring role to a different model family from the Student and Patient generators. Narrative recommendations were generated by a separate Feedback agent using Claude Haiku 4.5 with a temperature of 0.2. Full model versions, providers, routing endpoints, temperatures, and token settings are reported in Table S14 in Multimedia Appendix 1.

At runtime, the system loaded the selected approach and Patient-agent profile, executed a fixed-length exchange loop, and then called the Evaluator agent on the full transcript to generate criterion-level scores. The Feedback agent then generated criterion-referenced narrative recommendations.

### Student-Agent Conditions

To evaluate internal discrimination, we engineered 3 Student-agent prompts for each approach: novice, intermediate, and expert. These profiles were prompt-defined competence levels intended to generate stylized benchmark transcripts rather than represent the full distribution of real trainee performance. The novice, intermediate, and expert profiles were used to create controlled behavioral contrasts. This allowed us to test whether the Evaluator was sensitive to increases in prompt-defined rubric adherence under standardized Patient-agent and session-length conditions.

Prompts were self-contained and encoded the communication style, language patterns, and modality-specific behaviors. Each simulated session was capped at 10 therapist-patient exchanges, in which one exchange was defined as 1 Student-agent turn followed by 1 Patient-agent response. This fixed length was selected a priori as a pragmatic standardization constraint to keep dialogue length constant across Patient-agent profiles and Student-agent conditions, and to approximate a brief, focused training encounter rather than a full psychotherapy session.

The novice profile represented low rubric adherence, characterized by directive communication, limited reflection, weak collaboration, poor agenda setting, premature advice, or technique use, and limited use of modality-specific strategies. The intermediate profile represented partial and inconsistent rubric adherence, with some appropriate MI or CBT behaviors but recurrent omissions or poorly timed interventions. This condition was added to avoid relying solely on maximally separated novice or expert endpoints. The expert profile represented a high-adherence benchmark, with consistent use of modality-concordant behaviors such as reflective listening, autonomy support, evocation, collaborative agenda setting, Socratic questioning, case formulation, and structured homework planning.

Student-agent profiles were prompt-engineered AI agents, not human learners. The novice, intermediate, and expert conditions were designed to create stylized benchmark transcripts with expected differences in MI- or CBT-consistent behaviors. These conditions were used to test the internal Evaluator’s sensitivity to prompt-defined competence levels. Student prompts for MI and CBT, including novice, intermediate, expert, and novice+feedback prompt variants, are provided in Tables S7–S12 in Multimedia Appendix 1.

To prevent application-level carryover between trials, each Patient-profile × Student-condition run was executed in an independent session state. For each run, the system initialized empty interview and evaluator message histories, loaded the selected Patient-agent profile and Student-agent prompt, and generated a new transcript without access to prior transcripts, scores, feedback, or dialogue histories from other runs. Static resources, including model weights, model configurations, rubric prompts, and profile files, were reused as experimental inputs; however, session-level conversational state was not reused.

### Human Rater Scoring and Reliability Analysis

A pragmatic human-rater benchmark was assembled to compare Evaluator-agent scores with independent human ratings. For each modality, 16 human raters scored the selected transcripts. The panel included 3 practicing physicians with clinical experience using MI and CBT, with a median of 5 years of relevant clinical practice. Thirteen additional independent raters were recruited through Prolific. All raters provided informed consent and were compensated at a rate equivalent to US $20 per hour. Each rater independently scored 10 MI sessions across 9 MITI or MISC-derived criteria and 10 CBT sessions across eleven CTRS-derived criteria. Raters used the study-specific MI scoring form and the CTRS-derived CBT scoring form without access to other raters’ scores or Evaluator-agent outputs. Source manuals and item descriptions were provided as conceptual guidance.

Agreement analyses used session × criterion cells as the unit of analysis: 90 observations for MI and 110 observations for CBT. The intraclass correlation coefficient (ICC) (2,1) was used for 2-way random-effects, absolute-agreement, and single-rating reliability. Percentile-bootstrap 95% CIs were computed by resampling session × criterion observations. Because an Evaluator-vs-mean-consensus ICC is not structurally equivalent to a human interrater ICC, we also computed Evaluator-vs-individual-rater ICCs.

### Prompt-Augmentation Sensitivity Analysis

To assess whether criterion-referenced feedback text altered subsequent Student-agent behavior, we conducted a secondary prompt-augmentation sensitivity analysis. For each Patient-agent profile, the novice Student agent first completed a 10-exchange session. The recommendation section from the feedback report was then extracted and appended to the same novice Student-agent prompt before we ran a second 10-exchange session as a new, independent session state with the same Patient-agent profile. The underlying novice prompt was otherwise unchanged. This analysis was designed to test system responsiveness to feedback text within the simulation pipeline.

### Exploratory Student-Agent Dispersion Analysis

As an exploratory proxy for generative consistency, we analyzed token-level log probabilities from Student-agent responses. For each Student-agent turn, token log probabilities were extracted during GPT-4.1-mini generation, clipped at −10 to reduce the influence of sentinel or near-zero-probability tokens, and averaged across tokens. The session-level Student-agent average log probability was then computed as the mean across the 10 Student-agent turns. For interpretability, this value was transformed to the geometric mean token probability: exp(the session average log probability) × 100. We summarized variability across Patient-agent profiles within each Student-agent condition using SD and IQR, with bootstrap CIs.

### Missing Data

Missingness was assessed for generated transcripts, Evaluator-assigned criterion scores, AnnoMI labels, and human-rater scores. No missing data were observed in the final analytic datasets; therefore, no imputation was performed.

### Data Analysis

Criterion-level scores were analyzed within MI (9 criteria, 1‐5 scale) and CBT (11 criteria, 0‐6 scale). Internal discrimination analyses compared novice, intermediate, and expert Student-agent conditions within Patient-agent profiles. Paired comparisons across Student-agent conditions and pre- or post-prompt-augmentation comparisons were conducted using 2-sided Wilcoxon signed-rank tests with a significance *α* level of .05. For criterion-level analyses, Benjamini-Hochberg false discovery rate (FDR) correction was applied within each modality and planned contrast family. Adjusted *q* values are reported for criterion-level comparisons, with *q*<0.05 considered statistically significant. Overall-average comparisons were treated as separate planned summary tests and are reported with their corresponding *P* values, absolute mean changes, and effect sizes. Effect sizes were reported as rank-biserial correlations.

For mean scores and mean changes, 95% CIs were estimated using nonparametric bootstrap resampling across Patient-agent profiles. For the AnnoMI external comparison, Evaluator-assigned global MI scores were compared with coarse metadata-derived high or low session-level quality labels. Scores less than or equal to the prespecified threshold were classified as low quality, and scores greater than the threshold were classified as high quality. Accuracy, precision, recall, and *F*_1_-score were computed with high quality as the positive class and reported with 95% CIs. Human-rater reliability analyses used ICC(2,1), weighted Cohen κ, mean within-cell SD, mean absolute error, root mean square error, Pearson correlation, and Spearman correlation, as applicable. All statistical analyses were performed using Python version 3.11. Nonparametric procedures were selected because rubric scores were ordinal and potentially nonnormally distributed.

### Ethical Considerations

This work was conducted as a technical feasibility and simulation-based benchmarking study of a multiagent LLM framework prior to future controlled educational or clinical evaluation studies. The simulation and secondary-data components used synthetic agent-generated transcripts and deidentified source materials. No real patients or real learners participated; no identifiable patient information was used; and no clinical decisions were informed by the study outputs. In accordance with the Tri-Council Policy Statement: Ethical Conduct for Research Involving Humans–TCPS 2 [33], the simulation and secondary-data components were determined not to require research ethics board review.

Human involvement was limited to the independent evaluation of synthetic or deidentified transcripts. Raters were informed about the purpose and procedures of the rating task, provided informed consent before participation, and were compensated at a rate equivalent to US $20 per hour. Raters did not interact with patients, learners, or clinical systems. No personal health information or sensitive clinical information was collected from raters. Rater data were stored and analyzed in deidentified form and reported only in aggregate. Formal Research Ethics Board review was not sought because this work was classified as minimal-risk technical feasibility and simulation-based benchmarking research. This classification was based on the use of synthetic or deidentified transcripts, the limited scope of human-rater involvement, the absence of patient or learner interaction, the absence of personal health information or sensitive participant data, deidentified analysis, and aggregate reporting only. Future studies designed to evaluate real learners, standardized or real patients, educational effectiveness, clinical performance, or identifiable participant information will undergo formal Research Ethics Board review prior to implementation.

## Results

### Overview of the Evaluation Framework

**Figure 1.**
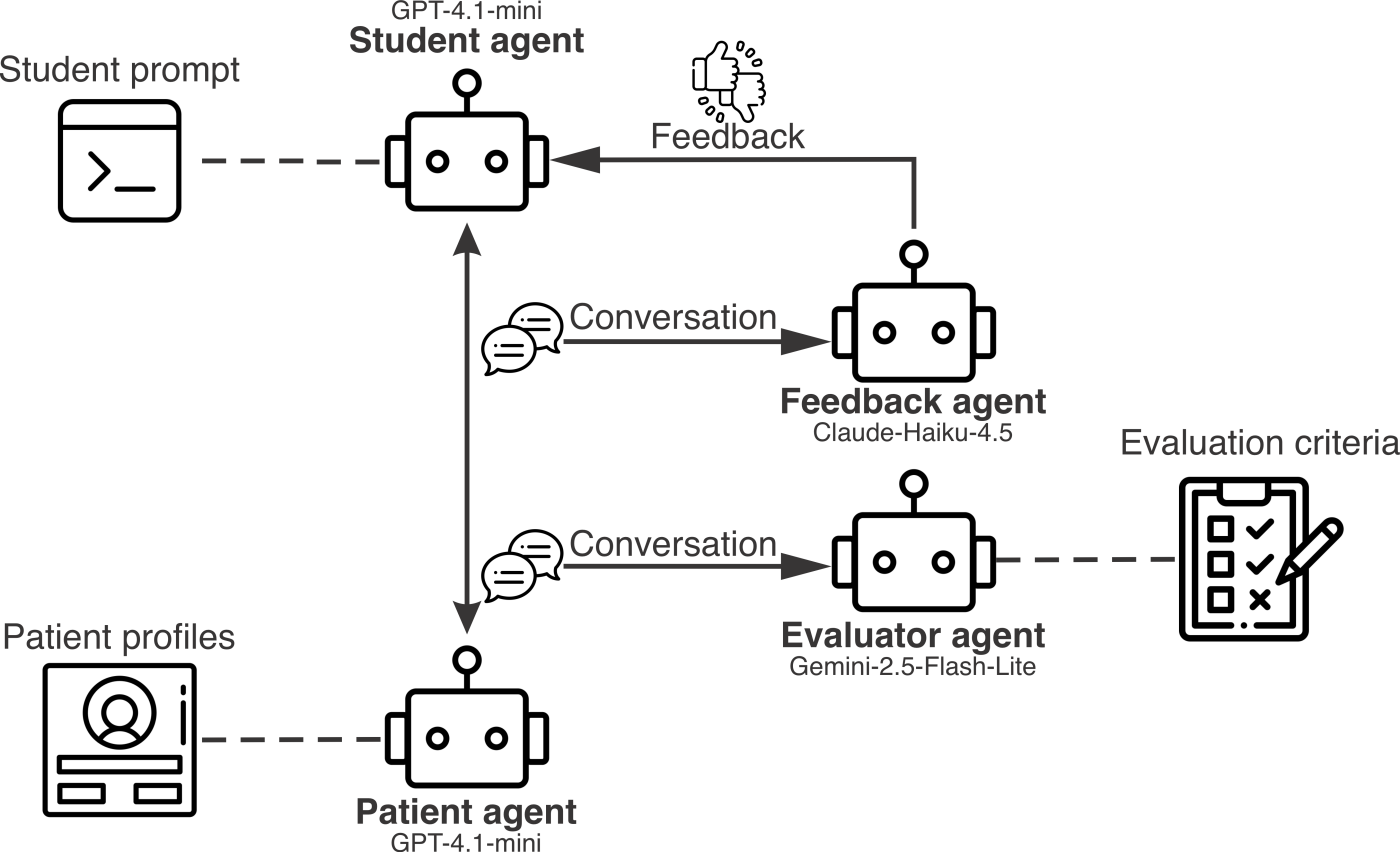
Multiagent large language model framework for stylized psychotherapy-training simulation and rubric-based evaluation. The system comprises role-separated Student, Patient, Evaluator, and Feedback agents coordinated within a structured dialogue workflow. The Student agent, instantiated with GPT-4.1-mini, conducts the synthetic psychotherapy encounter based on a predefined prompt. The Patient agent, also instantiated with GPT-4.1-mini, enacts a structured Patient-agent profile and generates case-consistent responses during the fixed-length dialogue. The Evaluator agent, instantiated with Gemini 2.5 Flash Lite, scores the completed transcript against predefined evaluation criteria. The Feedback agent, instantiated with Claude Haiku 4.5, generates criterion-referenced narrative recommendations based on the transcript, scoring criteria, and the scored evaluation output. Feedback text could be appended to the novice Student-agent prompt in a secondary prompt-augmentation sensitivity analysis. Full model configurations, temperatures, and routing details are reported in Table S14 in Multimedia Appendix 1.

The evaluation consisted of fixed-length synthetic encounters between a Student agent and structured Patient agents in 2 psychotherapeutic approaches, MI and CBT, with Student-agent performance scored by an Evaluator agent using predefined rubric criteria (Figure 1). For MI, the Evaluator used a study-specific, 9-criterion MITI or MISC–derived global-rating form on a 1 to 5 scale (Table S4 in Multimedia Appendix 1). For CBT, the Evaluator applied an 11-item CTRS–derived form on a 0 to 6 scale (Table S4 in Multimedia Appendix 1). All sessions used the fixed 10-exchange format defined in the “Methods” section. Scores were averaged across 133 MI and 102 CBT Patient-agent profiles for each of the 3 prompt-defined Student-agent competence profiles: novice, intermediate, and expert.

### Internal Discrimination Across Prompt-Defined Competence Levels

**Figure 2.**
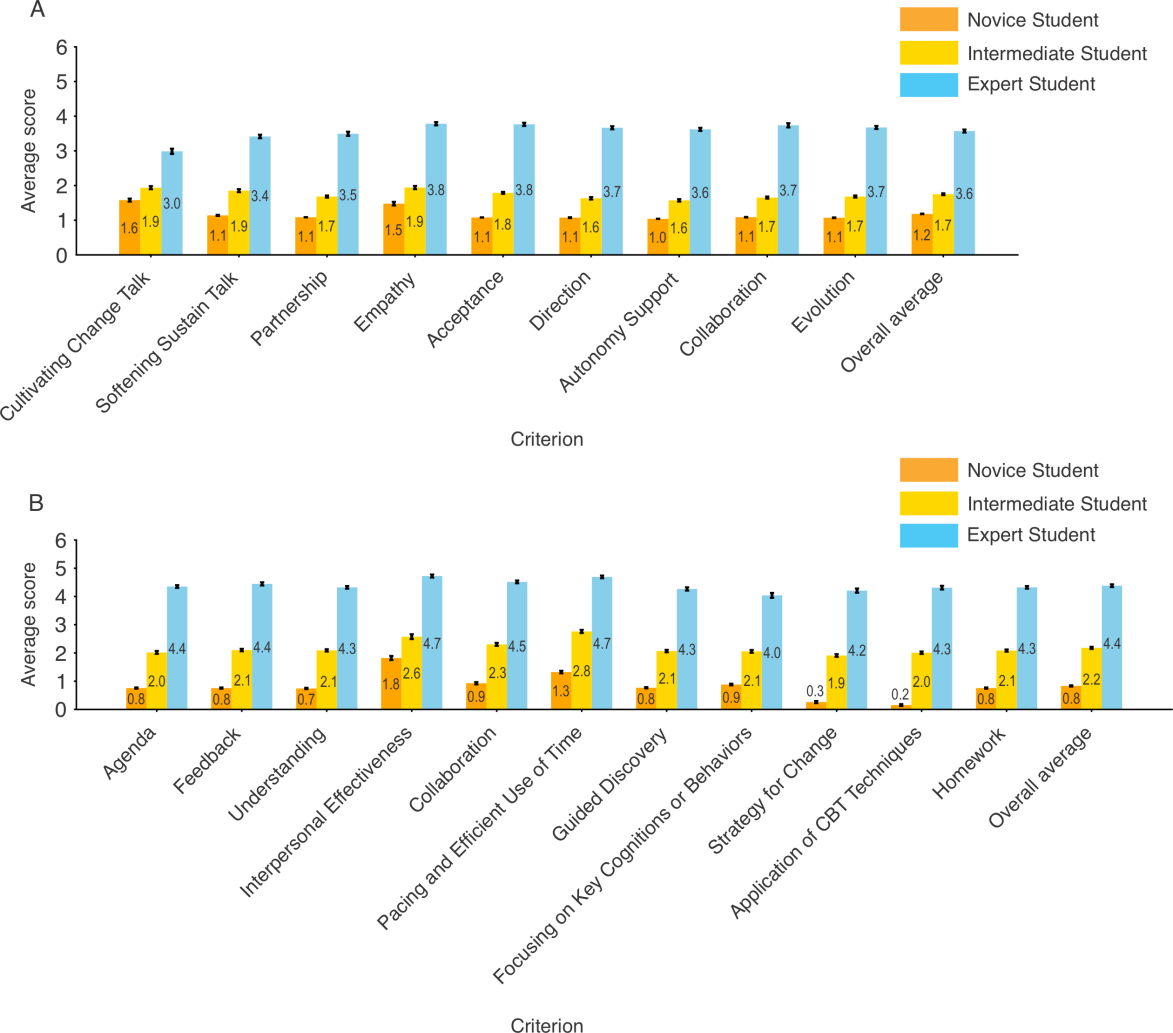
Evaluator-scored performance across prompt-defined novice, intermediate, and expert Student-agent profiles. (A) Average criterion-level scores for motivational interviewing (MI) across 133 simulated Patient-agent profiles, evaluated using a study-specific Motivational Interviewing Treatment Integrity or Motivational Interviewing Skill Code (MITI/MISC)–derived 1 to 5 global-rating form. (B) Average criterion-level scores for cognitive behavioral therapy (CBT) across 102 simulated Patient-agent profiles, evaluated using a Cognitive Therapy Rating Scale (CTRS)–derived 0 to 6 scoring form. Bars show mean scores and error bars show the SE of the mean. Scores demonstrate graded separation across prompt-engineered competence levels. Criterion-level significance testing used paired Wilcoxon signed-rank tests with Benjamini-Hochberg false discovery rate correction within each modality and planned contrast; adjusted *q* values were used to determine significance. These analyses evaluate internal scoring sensitivity across stylized prompt-defined Student-agent profiles.

Evaluator scores showed graded increases across the 3 prompt-defined student-agent competence conditions. For MI, the overall mean score increased from 1.18 (95% CI 1.16‐1.20) for the novice profile to 1.75 (95% CI 1.69‐1.81) for the intermediate profile and to 3.57 (95% CI 3.48‐3.66) for the expert profile (Figure 2A). For CBT, the overall mean score increased from 0.83 (95% CI 0.78‐0.88) to 2.18 (95% CI 2.10‐2.26) and 4.38 (95% CI 4.28‐4.48) for the intermediate and expert profiles, respectively (Figure 2B). All criterion-level planned contrasts across student-agent competence conditions remained significant after Benjamini-Hochberg false discovery rate correction (*q*<0.05). These findings support the Evaluator agent’s sensitivity to expected differences across stylized prompt-defined competence levels, but should be interpreted as an internal scoring-sensitivity benchmark rather than evidence of real-world learner assessment.

### External Comparison Using AnnoMI Session-Level Quality Labels

**Figure 3.**
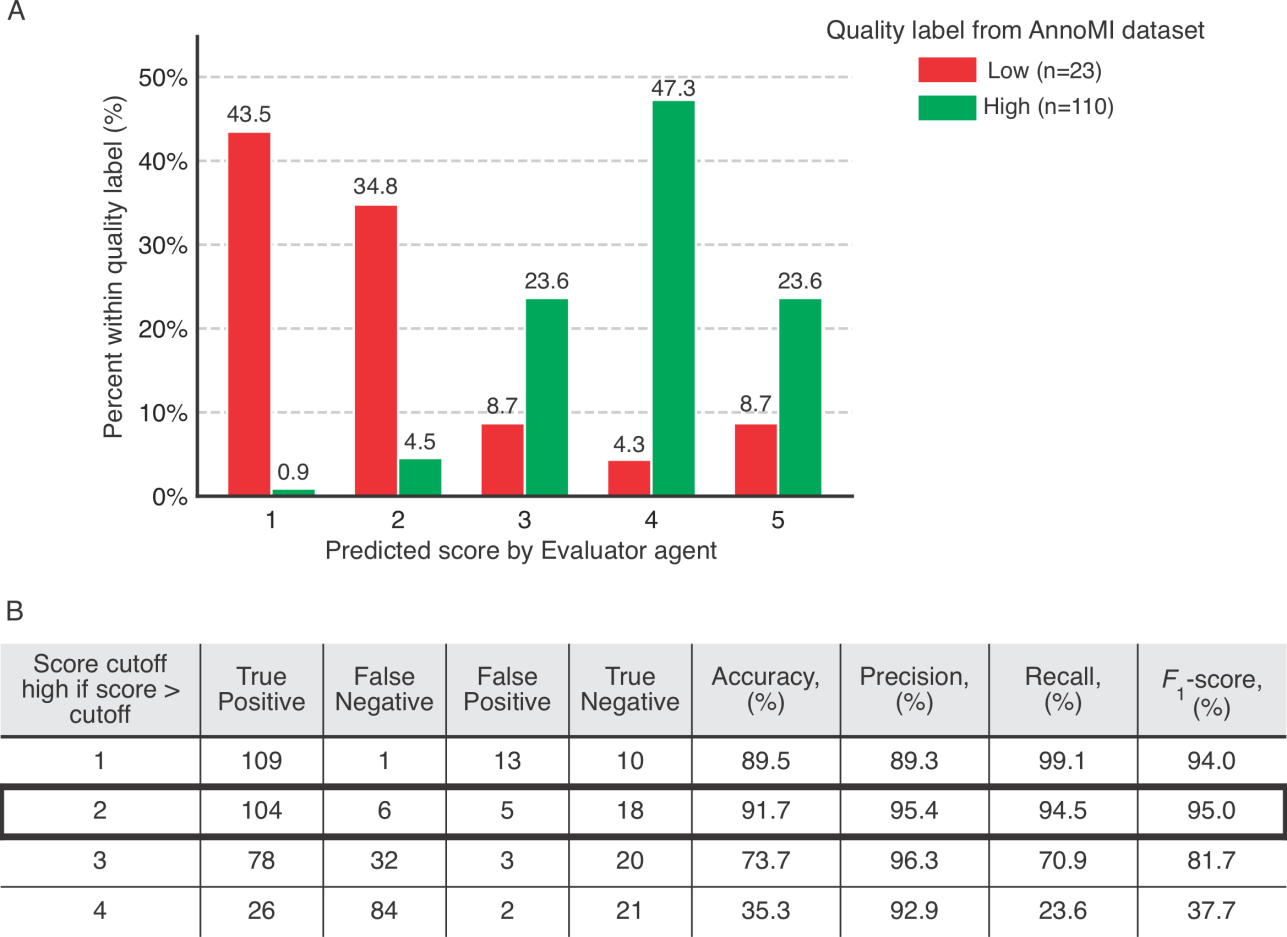
External comparison of Evaluator-agent motivational interviewing scores with annotated motivational interviewing (AnnoMI) metadata-derived quality labels. (A) Distribution of Evaluator-assigned motivational interviewing scores stratified by metadata-derived high or low session-level quality labels in the AnnoMI corpus. Bars show the percentage of sessions within each quality-label group receiving each score: low label (n=23) and high label (n=110). (B) Classification performance across Evaluator-score thresholds. Scores less than or equal to the threshold were classified as low quality, and scores greater than the threshold were classified as high quality. Performance metrics were computed with high quality as the positive class. A threshold of 2 yielded accuracy of 91.7%, precision of 95.4%, recall of 94.5%, and *F*_1_-score of 95.0%. These metrics reflect alignment with coarse metadata-derived session-level labels.

We next examined whether Evaluator scores aligned with external session-level quality labels in the AnnoMI corpus (Figure 3). AnnoMI contains expert utterance-level annotations [34], but this analysis used only the coarse high or low session-level quality labels associated with the source videos. Metadata-labeled high-quality sessions clustered primarily at Evaluator scores of 3 to 5, whereas metadata-labeled low-quality sessions clustered primarily at scores of 1 to 2. Using a threshold of 2, where scores ≤2 were classified as low quality and scores >2 were classified as high quality, yielded 91.7% accuracy, 95.4% precision, 94.5% recall, and an *F*_1_-score of 95.0%.

### Agreement Between Evaluator Scores and Human Raters

**Figure 4.**
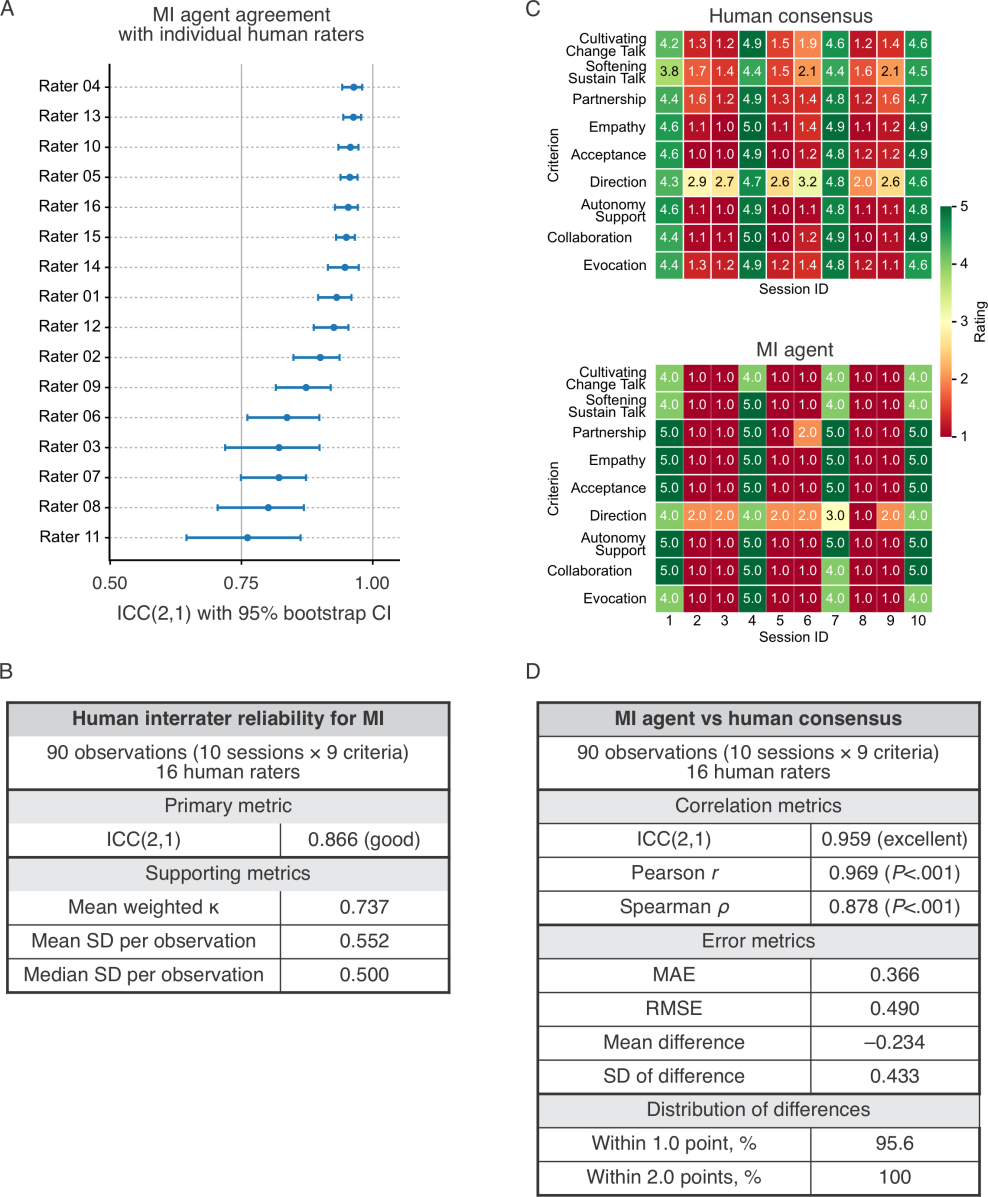
Human rater reliability and Evaluator-agent agreement with individual raters and the human-rater consensus for motivational interviewing (MI) scoring. Sixteen human raters per modality independently scored selected transcripts using the study-specific scoring forms. (A) Evaluator-agent agreement with each individual human rater for motivational interviewing (MI), shown as the intraclass correlation coefficient (ICC; 2,1) with 95%-bootstrap CIs between 90 session × criterion observations. (B) Human interrater reliability for MI across 16 human raters. (C) MI heatmaps comparing the mean human-rater consensus with Evaluator-agent scores. (D) Evaluator-agent agreement with the MI human-rater consensus. MAE: mean absolute error; RMSE: root mean square error.

**Figure 5.**
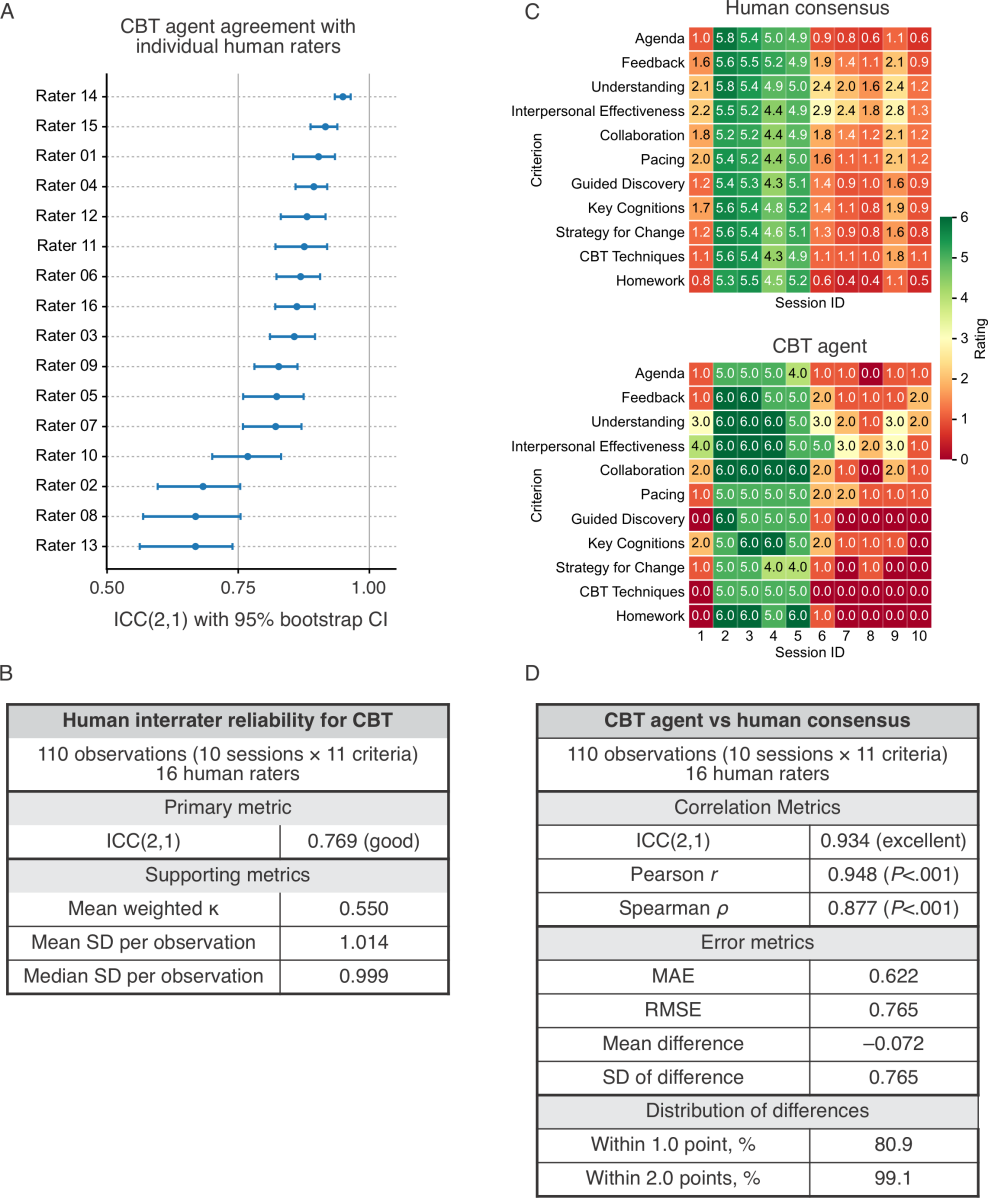
Human rater reliability and Evaluator-agent agreement with individual raters and the human-rater consensus for cognitive behavioral therapy (CBT) scoring. Sixteen human raters per modality independently scored selected transcripts using the study-specific scoring forms. (A) Evaluator-agent agreement with each individual human rater for cognitive behavioral therapy (CBT), shown as ICC(2,1) with 95% percentile-bootstrap CIs across 110 session × criterion observations. (B) Human interrater reliability for CBT across 16 human raters. (C) CBT heatmaps comparing the mean human-rater consensus with Evaluator-agent scores. (D) Evaluator-agent agreement with the CBT human-rater consensus. ICC: intraclass correlation coefficient; MAE: mean absolute error; RMSE: root mean square error.

We compared Evaluator-agent scores with ratings from a pragmatic 16-rater human benchmark (Figures 4 and 5). Raters independently scored 10 selected MI sessions across 9 MITI- or MISC-derived criteria and 10 selected CBT sessions across 11 CTRS-derived criteria, yielding 90 and 110 session × criterion observations, respectively. Session-level heatmaps showed broadly similar, though not identical, rating patterns across the human-rater consensus and Evaluator-agent scores in both approaches.

Human interrater reliability was good for MI, with an ICC(2,1) of 0.866 (95% CI 0.825‐0.898) across 90 session × criterion observations, a mean weighted κ of 0.737, a mean SD per observation of 0.552, and a median SD per observation of 0.500. For CBT, human interrater reliability was an ICC(2,1) of 0.769 (95% CI 0.732‐0.799) across 110 observations, a mean weighted κ of 0.550, a mean SD per observation of 1.014, and a median SD per observation of 0.999.

Evaluator-vs-human-consensus agreement was high for MI, with ICC(2,1) of 0.959 (95% CI 0.940‐0.973), Pearson *r* of 0.969, Spearman ρ of 0.878, mean absolute error (MAE) of 0.366, and root mean square error (RMSE) of 0.490. For CBT, Evaluator-vs-human-consensus agreement was ICC(2,1) of 0.934 (95% CI 0.913‐0.950), Pearson *r* of 0.948, Spearman ρ of 0.877, MAE of 0.622, and RMSE of 0.765.

Because agreement with an averaged human consensus is not directly comparable with interrater reliability among individual humans, we additionally computed Evaluator-vs-individual-rater ICCs. These ranged from 0.761 to 0.964 for MI and from 0.669 to 0.950 for CBT. In leave-one-human-out analyses, individual human raters compared with the mean of the remaining human raters showed median ICCs of 0.944 for MI and 0.877 for CBT. These analyses contextualize the Evaluator agent’s agreement with both individual raters and consensus ratings.

### Impact of Evaluator Feedback on Novice Performance

**Figure 6.**
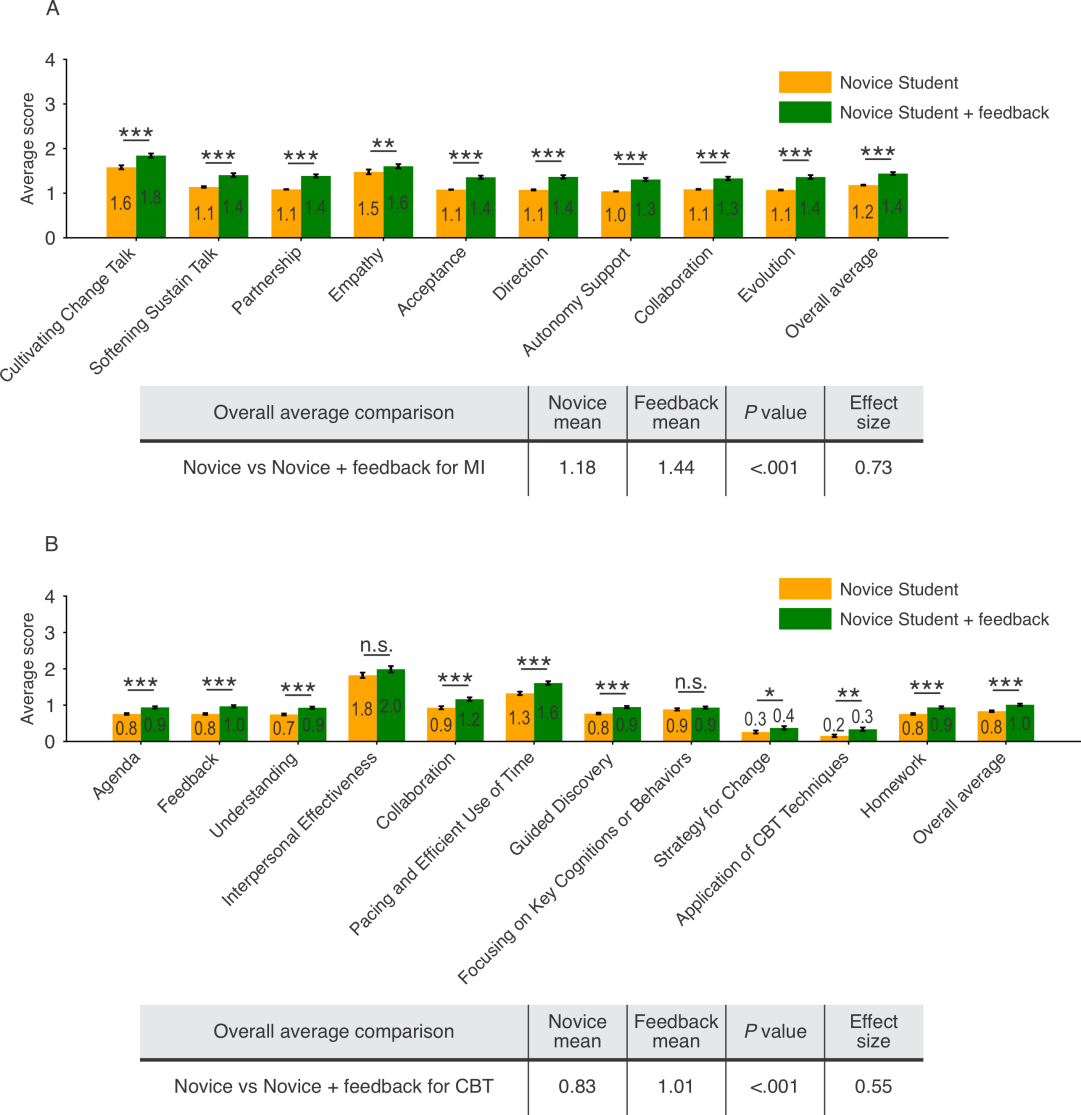
Prompt-augmentation sensitivity analysis using feedback text appended to the novice Student-agent prompt. (A) Motivational interviewing (MI) criterion-level scores before and after appending feedback recommendations to the novice Student-agent prompt across 133 Patient-agent profiles. The overall average increased from 1.18 to 1.44, corresponding to an absolute change of +0.26 points (*P*<.001) and a rank-biserial effect size of 0.73. All 9 MI criteria showed significant increases after false discovery rate correction. (B) Cognitive behavioral therapy (CBT) criterion-level scores before and after feedback-text prompt augmentation across 102 Patient-agent profiles. The overall average increased from 0.83 to 1.01, corresponding to an absolute change of +0.18 points (*P*<.001) and a rank-biserial effect size of 0.55. A total of 9 of 11 CBT criteria showed significant increases after false discovery rate correction. Asterisks denote Benjamini-Hochberg false discovery rate–adjusted criterion-level significance: *q<0.05, **q<0.01, ***q<0.001; n.s.=not significant after false discovery rate correction. Overall-average *P* values in the tables correspond to separate planned paired Wilcoxon signed-rank tests.

In the prompt-augmentation sensitivity analysis, appending feedback recommendations to the novice Student-agent prompt produced modest score shifts in subsequent synthetic sessions (Figure 6). For MI, the overall average increased from 1.18 to 1.44, corresponding to an absolute mean change of +0.26 points (95% CI 0.19‐0.33; *P*<.001; rank-biserial effect size=0.73). All 9 MI criteria showed significant increases after false discovery rate correction (Figure 6A). For CBT, the overall average increased from 0.83 to 1.01, corresponding to an absolute mean change of +0.18 points (95% CI 0.11‐0.25; *P*<.001; rank-biserial effect size=0.55). A total of 9 of 11 CBT criteria showed significant increases after false discovery rate correction; Interpersonal Effectiveness and Focusing on Key Cognitions or Behaviors were not significant (Figure 6B). These findings indicate that feedback-text prompt augmentation shifted evaluator-assigned scores within the synthetic pipeline.

Narrative feedback outputs were criterion-referenced and score-linked. In representative MI outputs, low scores in Acceptance, Empathy, Partnership, Autonomy Support, and Evocation were accompanied by explanations citing judgmental language, lack of reflective listening, directive advice, limited collaboration, and failure to elicit change talk. Corresponding recommendations emphasized avoiding confrontation, using complex reflections, eliciting patient values, and supporting patient choice. In representative CBT outputs, weak Agenda, Conceptualization, Guided Discovery, and Homework scores were paired with recommendations for collaborative agenda setting, psychoeducation, Socratic questioning, and structured between-session practice. These examples illustrate structural alignment between criterion scores, explanations, and recommendations.

### Exploratory Response-Consistency Analysis

**Figure 7.**
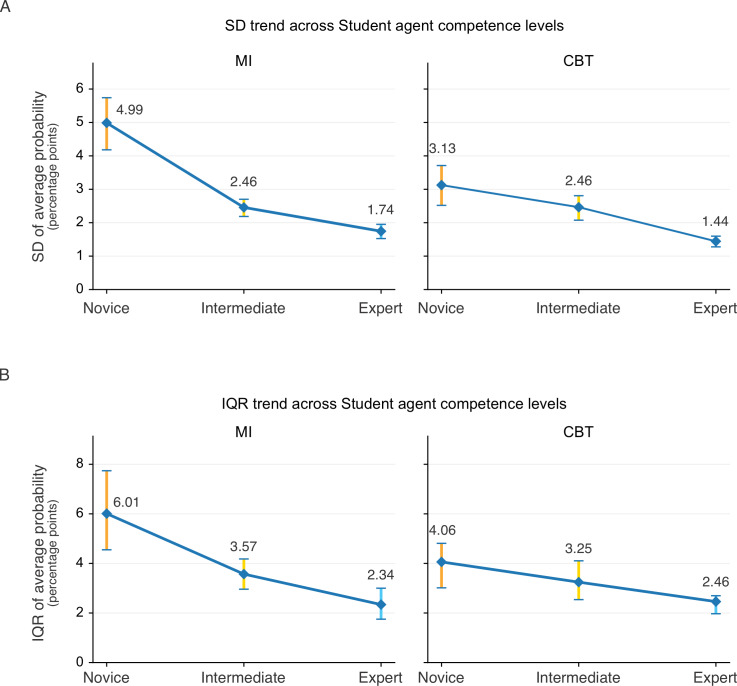
Exploratory Student-agent log-probability dispersion across prompt-defined competence levels. For each simulated session, Student-agent token log probabilities were averaged across generated tokens and turns, then transformed into geometric mean token probability percentages. (A) SD of the session-level average probability across Patient-agent profiles for novice, intermediate, and expert Student-agent conditions in motivational interviewing (MI) and cognitive behavioral therapy (CBT). (B) IQR of the same measure. Error bars indicate bootstrap CIs. Lower dispersion indicates more consistent model response probabilities across Patient-agent profiles. Expert Student-agent responses showed lower dispersion than novice responses in both MI and CBT.

Figure 7 shows the dispersion of Student-agent response log-probability-derived average token probability across Patient-agent profiles. Dispersion decreased across prompt-defined competence levels. For MI, the SD decreased from 4.99 percentage points (95% CI 4.19‐5.77) for the novice profile to 2.46 (95% CI 2.19‐2.70) for the intermediate profile to 1.74 (95% CI 1.53‐1.95) for the expert profile; the corresponding IQR decreased from 6.01 (95% CI 4.50‐7.76) to 3.57 (95% CI 2.96‐4.22) to 2.34 (95% CI 1.75‐3.00). For CBT, the SD decreased from 3.13 (95% CI 2.51‐3.70) to 2.46 (95% CI 2.08‐2.82) to 1.44 (95% CI 1.28‐1.60), and the IQR decreased from 4.06 (95% CI 2.98‐4.80) to 3.25 (95% CI 2.55‐4.10) to 2.46 (95% CI 1.97‐2.69). These findings suggest that higher-adherence Student-agent prompts produced more constrained and consistent responses across Patient-agent profiles. This exploratory analysis is an indirect proxy for generative consistency and does not directly measure hallucination, clinical realism, or safety.

A prototype user interface was developed to demonstrate how the multiagent workflow could be presented to end users, including approach selection, text- or voice-based practice, and display of criterion-level scores with narrative feedback (Figure S1 in Multimedia Appendix 1). This interface was not used in the present evaluation analyses and should be interpreted only as an implementation demonstration. Overall, the results provide early-stage evidence that a rubric-guided Evaluator agent can score stylized synthetic MI and CBT transcripts along expected prompt-defined competence gradients, align with a preliminary external comparison and a pragmatic human-rater benchmark, and show modest score shifts after feedback-text prompt augmentation.

## Discussion

### Principal Findings: Scalable and Interpretable Assessment of Psychotherapeutic Competence

Integration of LLMs into health professions education may transform how trainees acquire and practice complex communication skills at scale [8,18,19,21,25]. In this preclinical, cross-sectional, simulation-based evaluation, we assessed a rubric-guided, multiagent LLM framework for stylized MI and CBT training simulations. The Evaluator agent scored synthetic transcripts in the expected direction across prompt-defined novice, intermediate, and expert Student-agent profiles; showed preliminary alignment with coarse AnnoMI session-level quality labels; aligned with a pragmatic human-rater benchmark; and showed modest score shifts after feedback-text prompt augmentation. The exploratory log-probability dispersion analysis further suggested that higher-adherence Student-agent prompts produced more constrained and consistent responses across Patient-agent profiles. By coupling standardized Patient simulations with an Evaluator that maps behaviors to established instruments, the system provides formative and summative signals that are interpretable within recognized competency frameworks [35]. Because the agents operate over explicit rubrics and supply narrative justifications, the approach is amenable to audit and standard setting and offers a path to consistent assessment across sites and cohorts [36,37].

### Interpretation and Comparison With Prior Work

The main innovation of this work is the role-separated architecture, in which Patient, Student, Evaluator, and Feedback agents serve distinct functions within one simulation workflow [25]. Prior LLM studies in health professions education have largely emphasized plausible simulated conversations or tutoring interactions [7,20]. This study extends that work by linking synthetic psychotherapy encounters to explicit scoring criteria, external comparison data, pragmatic human-rater agreement, and criterion-referenced feedback outputs [16,17]. The contribution is therefore not that the system reproduces real psychotherapy, but that it provides an auditable framework for testing whether an LLM-based Evaluator responds to controlled, rubric-relevant differences in synthetic transcripts.

The internal discrimination analysis is important because a scoring system intended for psychotherapy training should, at minimum, assign higher scores when transcripts contain more rubric-concordant therapist behaviors [38]. The graded pattern across novice, intermediate, and expert Student-agent profiles supports this internal scoring sensitivity. However, these Student-agent profiles were engineered competence archetypes and should not be interpreted as empirical representations of medical students, therapists in training, or expert clinicians. Future studies must test whether similar scoring patterns hold in human learners across defined training stages [8].

The human-rater comparison provides a complementary benchmark for interpreting the Evaluator’s scores. Although the rater panel was not composed of calibrated fidelity coders, Evaluator scores aligned closely with the average human-rater consensus and showed similar patterns when compared with individual raters. This suggests that, within the constrained setting of stylized synthetic transcripts and study-specific scoring forms, the Evaluator’s rubric-based judgments approximated the ratings assigned by independent human raters. This finding is consistent with prior work suggesting that LLM-based evaluators can approximate human ratings [39,40]. However, this agreement should not be interpreted as evidence of superiority over human raters or as a substitute for validation against trained fidelity coders.

Some criteria, particularly Empathy and Autonomy Support, require careful interpretation in LLM-agent simulations. In this study, Empathy was scored as observable language indicating understanding, validation, and reflective response to the patient’s stated experience. Autonomy Support was scored as noncoercive, choice-supporting language consistent with MI principles. These are transcript-level behavioral ratings, not evidence that the LLM possesses affective empathy, clinical attunement, or the capacity to form a therapeutic relationship. Prior work on LLM empathy has similarly emphasized the distinction between responses perceived as empathic and genuine human empathy [41]. We therefore interpret these scores as evidence that the Evaluator detected rubric-concordant language patterns in synthetic transcripts, not as evidence of authentic empathic capacity.

The narrative feedback component also requires cautious interpretation. Feedback outputs were criterion-referenced and structurally aligned with scores, explanations, and recommendations. This structure is useful for formative simulation design because it makes the basis for feedback explicit and reviewable [20,42]. However, narrative feedback alignment was assessed qualitatively and illustratively in this study; it was not independently validated by blinded experts for accuracy, clinical appropriateness, safety, or educational utility [35,37]. Similarly, the prompt-augmentation analysis showed that appending feedback text shifted subsequent Student-agent scores, but it does not demonstrate feedback efficacy, human learning, or skill transfer [42].

### Limitations and Future Directions

A central limitation is that the simulated transcripts were stylized outputs of prompt-engineered agents rather than interactions involving real patients or human trainees. We did not compare generated dialogues with real psychotherapy sessions, and we did not test whether Patient or Student agents reproduced the behavior of actual patients or trainees. This design supported controlled benchmarking but limited assessment of flexible adaptation to complex, comorbid, culturally nuanced, or diagnostically ambiguous presentations [43,44]. Similarly, the fixed 10-exchange session length standardized exposure across conditions but restricted observation of longer-form psychotherapy processes such as alliance development, rupture repair, homework review, and multisession change [45,46]. Accordingly, the findings should be interpreted as evidence that the Evaluator can score controlled synthetic examples along expected rubric-defined gradients, not as evidence of ecological validity or readiness for summative assessment of real learners [8,47].

The external and human-rating benchmarks also have important limitations. Although AnnoMI contains expert utterance-level annotations [31], this study used only coarse high or low session-level quality labels derived from video metadata and source context; these labels are not equivalent to expert transcript-level MI fidelity ratings. In addition, because AnnoMI is publicly available and predates the Evaluator model’s knowledge cutoff, training-data contamination cannot be excluded [48]. The human-rating analysis should likewise be interpreted as a pragmatic benchmark rather than a fidelity-coding reference standard. Although the rater panel provided useful comparison data, raters were not formally calibrated MITI, MISC, or CTRS fidelity coders. Future validation should therefore include trained and calibrated fidelity coders, predefined reliability thresholds, full fidelity-coding procedures, and prospectively collected transcripts from human trainees interacting with standardized or real patients.

The prompt-augmentation and feedback analyses should not be interpreted as evidence of educational efficacy. No human learner received feedback, practiced with the system, demonstrated skill retention, or transferred skills to a new clinical task. Instead, the postfeedback Student-agent prompt contained feedback recommendations appended to an otherwise novice prompt, meaning that observed score shifts may reflect LLM instruction-following or prompt sensitivity rather than learning [49,50]. Narrative feedback outputs were criterion-referenced and structurally aligned with scores, explanations, and recommendations, but they were not independently validated by blinded experts for accuracy, clinical appropriateness, safety, or educational utility. Future studies should test feedback prospectively in human trainees, using independent feedback delivery, delayed reassessment, blinded expert ratings, and evaluation on unseen standardized-patient or real-patient encounters [51-53].

Finally, several implementation and safety limitations remain. LLMs are susceptible to hallucination, misinterpretation, unsafe recommendations, bias, and calibration drift [54,55]. This study did not formally adjudicate or quantify failure events such as hallucinated clinical details, internally inconsistent Patient-agent responses, or clinically inappropriate recommendations. The exploratory log-probability dispersion analysis provides only an indirect proxy for generative consistency and does not directly measure clinical realism, therapeutic safety, or fidelity to patient complexity. Because the simulations were text-only, the system could not evaluate tone, pacing, prosody, facial affect, gestures, silence, turn timing, or other nonverbal and paralinguistic behaviors that contribute to alliance and clinical communication [7,56,57]. Future versions should incorporate structured safety monitoring, expert adjudication of failure modes, multimodal inputs such as audio or video, and validation by domain-specific clinical experts [56]. Model selection is also implementation-dependent: using different model families reduces direct same-model self-evaluation, but it does not eliminate shared pretraining exposure, model bias, or calibration drift [58,59]. These issues should be addressed before the system is used beyond low-stakes formative simulation or research settings.

### Conclusions

This proof-of-concept study suggests that a rubric-guided multiagent LLM framework can score stylized synthetic MI and CBT transcripts along expected prompt-defined competence gradients and align with preliminary external and pragmatic human-rater benchmarks. The study is innovative in separating synthetic patient simulation, synthetic learner behavior, rubric-based scoring, and narrative feedback generation within a single auditable workflow. It differs from prior LLM simulation studies by moving beyond face-valid dialogue generation toward structured, criterion-linked evaluation. The findings support further development of scalable simulation tools for psychotherapy training research, fidelity-methods development, and low-stakes formative practice.

## Supplementary material

10.2196/92964 Multimedia Appendix 1 Supplementary figure and tables.

10.2196/92964 Checklist 1 DECIDE-AI checklist.

## Abbreviations:

AnnoMI: annotated motivational interviewing
CBT: cognitive behavioral therapy
CTRS: Cognitive Therapy Rating Scale
DECIDE-AI: Developmental and Exploratory Clinical Investigations of Decision Support Systems Driven by AI
*DSM-5*: *Diagnostic and Statistical Manual of Mental Disorders, Fifth Edition*
FDR: false discovery rate
ICC: intraclass correlation coefficient
LLM: large language model
MAE: mean absolute error
MI: motivational interviewing
MISC: Motivational Interviewing Skill Code
MITI: Motivational Interviewing Treatment Integrity
RMSE: root mean square error
