# Supplementary material for: Multiagent Large Language Model Framework for Psychotherapy Fidelity Assessment in Motivational Interviewing and Cognitive Behavioral Therapy Training: Cross-Sectional, Simulation-Based Evaluation Study
Source: JMIR Med Educ. 2026 Aug 18;12:e92964. doi: 10.2196/92964 (PMC13483752; doi:10.2196/92964)
Supplement: Supplementary file 1 [file mededu-v12-e92964-s001.docx]

**Supplementary Figure 1. Example user-interface views from the multi-agent simulation platform.** **(A)** Session initiation screen showing patient case summary and Evaluator introduction prior to starting a simulated MI encounter. **(B)** Therapist-patient dialogue interface allowing typed or spoken responses during the simulated session. **(C)** Interactive Evaluator view providing narrative, criterion-referenced feedback based on the completed transcript. **(D)** Alternative detailed feedback view illustrating item-level scoring and explanations aligned with MI fidelity criteria. These interface examples are provided for demonstration purposes and were not used in any validation analyses.

**Supplementary Table 2. Example MI Patient-agent system prompt.** The profile is organized into structured prompt fields defining the patient role, clinical context, MI-relevant ambivalence and change-talk content, behavioral response guidance, and simulation constraints used to instantiate the Patient agent. The MI simulation-prompt fields were derived from the manually constructed MI profile template, MITI/MISC-informed constructs, and common behavior-change role-play scenarios. They were used to guide Patient-agent expression of ambivalence, sustain talk, change talk, readiness to change, emotional tone, and disclosure level.

**Supplementary Table 3. Example CBT Patient-agent system prompt. The profile is organized into structured prompt fields defining the patient role, presenting problem, symptoms, cognitive and behavioral content, history, diagnosis, and response guidance used to instantiate the Patient agent. The CBT simulation-prompt fields were derived from DSM-5 Clinical Cases case content and reformatted into Patient-agent response guidance, including symptom presentation, cognitive/behavioral content, emotional tone, and disclosure style**.

**Supplementary Table 4. MI evaluation criteria and descriptions.** Criteria used by the Evaluator agent to assess Student performance in the MI. The rubric integrates items from the Motivational Interviewing Treatment Integrity (MITI 4.2.1) and Motivational Interviewing Skill Code (MISC 2.5) manuals, each rated on a 1–5 Likert scale. Descriptions summarize the behavioral anchors defining how the counselor’s behavior is rated for each domain.

**Supplementary Table 5. CBT evaluation criteria and descriptions.** Criteria used by the Evaluator agent to score Student performance in the CBT. The rubric is based on CTRS and comprises eleven items rated on a 0–6 Likert-like scale. Descriptions reflect behavioral anchors for high-quality therapeutic practice across different domains. Together, these criteria capture the structure, process, and relational dimensions that define competent CBT delivery within the simulated psychotherapy encounters.

**Supplementary Table 6. MI Evaluator agent system prompt.** System prompt used to instantiate the Evaluator agent for MI scoring. The prompt provides structured instructions on how to review a simulated interview, deliver feedback, and apply criteria consistently. It defines required evaluation domains and guides the agent to produce specific, actionable, and evidence-based feedback aligned with MI principles.

**Supplementary Table 7. CBT Evaluator agent system prompt.** System prompt used to instantiate the Evaluator agent for CBT scoring. The prompt provides structured guidance on reviewing simulated CBT interviews, delivering feedback, and applying CTRS-based evaluation domains. It defines expectations for assessing applicable criteria, ensuring that the agent produces consistent, structured, and actionable feedback aligned with CBT competency standards.

**Supplementary Table 8. MI novice Student agent system prompt.** System prompt defining the behaviour of the novice MI Student agent. The prompt specifies a clinician persona lacking formal MI training and outlines characteristic errors such as directive communication, limited empathy, minimal exploration of ambivalence, and reliance on closed questions. It includes explicit behavioural and language constraints to ensure consistent enactment of novice-level MI performance during simulated encounters.

**Supplementary Table 9. MI novice Student + feedback system prompt.** System prompt used to instantiate the novice MI Student agent after incorporating Feedback agent’s recommendations. This prompt retains the behavioural and language constraints characteristic of novice MI performance while appending targeted recommendations from the Feedback agent during the subsequent simulated session.

**Supplementary Table 10. MI expert Student agent system prompt.** System prompt defining the behaviour of the expert MI Student agent. The prompt specifies high-fidelity MI practice characterised by partnership, empathy, and autonomy support. It outlines expert behavioural patterns throughout the session, ensuring a stable and exemplary MI performance during simulated encounters.

**Supplementary Table 11. CBT novice Student agent system prompt.** System prompt defining the behaviour of the novice CBT Student agent. The prompt specifies a clinician persona with limited understanding of CBT principles, characterised by unstructured interactions, generic encouragement, personal anecdotes, and minimal agenda setting. It outlines consistent novice-level errors, including avoidance of guided discovery, failure to explore thoughts-feelings-behaviours links, and omission of homework planning, thereby ensuring reproducibly poor CBT performance during simulated encounters.

**Supplementary Table 12. CBT novice Student + feedback system prompt.** System prompt defining the behaviour of the novice CBT Student agent after incorporating Feedback agent’s recommendations. The prompt preserves the novice-level behavioural and language constraints characteristic of unstructured, non-CBT-aligned interviewing, while appending targeted recommendations by the Feedback agent during the subsequent simulated session.

**Supplementary Table 13. CBT expert Student agent system prompt.** System prompt defining the behaviour of the expert CBT Student agent. The prompt specifies high-fidelity CBT interviewing characterised by collaborative agenda setting, empathy, structured guided discovery, and focus on key cognitions and behaviours. It outlines expert behavioural patterns to ensure consistently skilled CBT performance during simulated encounters.

**Supplementary Table 14. Example narrative feedback from the Feedback agent for MI and CBT sessions. (A)** Illustrative feedback provided by the Feedback agent following an MI session, highlighting specific behaviour-change targets (e.g., adopting a partnership stance, evoking change talk, increasing complex reflections) with concrete examples and practice suggestions. **(B)** Corresponding feedback for a CBT session, focusing on session structure (agenda setting), psychoeducation and conceptualisation, and the use of guided discovery to address automatic thoughts.

**Supplementary Table 15. Model configurations for the multi-agent simulation framework.** LLM configurations used for each agent in the multi-agent simulation framework. To mitigate circularity, Student and Patient agents were instantiated with GPT-4.1 mini (OpenAI), while the Evaluator agent employed Gemini 2.5 Flash-Lite (Google) and the Feedback agent used Claude Haiku 4.5 (Anthropic). The table lists provider information, knowledge-cutoff dates, maximum input and output token capacities, and approximate pricing per one million tokens for input, cached input, and output text as of April 2026.
