# Supplementary material for: Multiagent Large Language Model Framework for Psychotherapy Fidelity Assessment in Motivational Interviewing and Cognitive Behavioral Therapy Training: Cross-Sectional, Simulation-Based Evaluation Study
Source: JMIR Med Educ. 2026 Aug 18;12:e92964. doi: 10.2196/92964 (PMC13483752; doi:10.2196/92964)
Supplement: Supplementary file 2 [file mededu-v12-e92964-s002.docx]

**Alignment of the study with the DECIDE-AI reporting guideline.** This table details how each recommended item was addressed in the methods, analysis, and reporting.
